# New insights in the genetic variant spectrum of *SLC34A2* in pulmonary alveolar microlithiasis; a systematic review

**DOI:** 10.1186/s13023-023-02712-7

**Published:** 2023-05-31

**Authors:** Åsa Lina M. Jönsson, Ole Hilberg, Ulf Simonsen, Jane Hvarregaard Christensen, Elisabeth Bendstrup

**Affiliations:** 1 Department of Clinical Genetics, Aarhus University Hospital, Aarhus, Denmark; 2 Department of Biomedicine, Aarhus University, Aarhus, Denmark; 3 Department of Regional Health Research, University of Southern Denmark, Odense, Denmark; 4 Department of Medicine, Lillebaelt Hospital, Vejle, Denmark; 5 Centre for Rare Lung Diseases, Department of Respiratory Diseases and Allergy, Aarhus University Hospital, Aarhus, Denmark

**Keywords:** Pulmonary alveolar microlithiasis, Interstitial lung disease, Pulmonary calcification, Genetic diseases, Inborn, *SLC34A2* variants, *SLC34A2* mutations, *SLC34A2*, Solute carrier family 34 (sodium phosphate), member 2 protein, human

## Abstract

Pulmonary alveolar microlithiasis (PAM) is a rare autosomal recessive lung disease caused by variants in the *SLC34A2* gene encoding the sodium-dependent phosphate transport protein 2B, NaPi-2b. PAM is characterized by deposition of calcium phosphate crystals in the alveoli. Onset and clinical course vary considerably; some patients remain asymptomatic while others develop severe respiratory failure with a significant symptom burden and compromised survival. It is likely that PAM is under-reported due to lack of recognition, misdiagnosis, and mild clinical presentation. Most patients are genetically uncharacterized as the diagnostic confirmation of PAM has traditionally not included a genetic analysis. Genetic testing may in the future be the preferred tool for diagnostics instead of invasive methods. This systematic review aims to provide an overview of the growing knowledge of PAM genetics. Rare variants in *SLC34A2* are found in almost all genetically tested patients. So far, 34 allelic variants have been identified in at least 68 patients. A majority of these are present in the homozygous state; however, a few are found in the compound heterozygous form. Most of the allelic variants involve only a single nucleotide. Half of the variants are either nonsense or frameshifts, resulting in premature termination of the protein or decay of the mRNA. There is currently no cure for PAM, and the only effective treatment is lung transplantation. Management is mainly symptomatic, but an improved understanding of the underlying pathophysiology will hopefully result in development of targeted treatment options. More standardized data on PAM patients, including a genetic diagnosis covering larger international populations, would support the design and implementation of clinical studies to the benefit of patients. Further genetic characterization and understanding of how the molecular changes influence disease phenotype will hopefully allow earlier diagnosis and treatment of the disease in the future.

## Background

Pulmonary alveolar microlithiasis (PAM) (OMIM #265100) is an autosomal recessive lung disease where calcium-phosphate concretions (microliths) are formed in the alveoli [1–3]. PAM was first named by the Hungarian physician Ludwig Puhr in 1933 [4]. It is caused by variants in the *SLC34A2* gene (Entrez Gene ID 10568) encoding the sodium-dependent phosphate transport protein 2B, NaPi-2b [5–7]. The protein belongs to the sodium-transporter family SLC34, which is involved in the inorganic phosphate (Pi) homeostasis [8]. The incidence of PAM is unknown. Less than 1200 patients are described in the literature, and most descriptions are from Asia and Europe. Both familial and sporadic cases are reported. In almost all families with PAM, transmission is reported to be horizontal. In the rare case of vertical transmission, this has always been a result of consanguinity [9]. Although almost all patients in the literature who have been genetically evaluated have pathogenic variants in *SLC34A2*, genetic testing is not part of the routine diagnostic evaluation. However, genetic investigation is increasingly recommended [1]. This review will provide an overview of PAM with a specific focus on underlying genetic aspects.

## Search strategy

A structured literature search for the genetic part of the review was performed according to preferred reporting items for systematic reviews and meta-analyses (PRISMA) 2009 guidelines [10]. The following online reference databases were used: Embase, PubMed, SCOPUS, Cochrane, and Web of Science. Searches were carried out in August 2022. The search terms used were 'pulmonary alveolar microlithiasis' AND 'SLC34A2'. Additionally, a search in The Human Gene Mutation Database (HGMD) Professional was performed in August 2022 (HGMD Professional 2022.2) [11]. Furthermore, additional articles were identified from reference lists of studies included in this review and from existing reviews.

### Study selection process

**Fig. 1:**
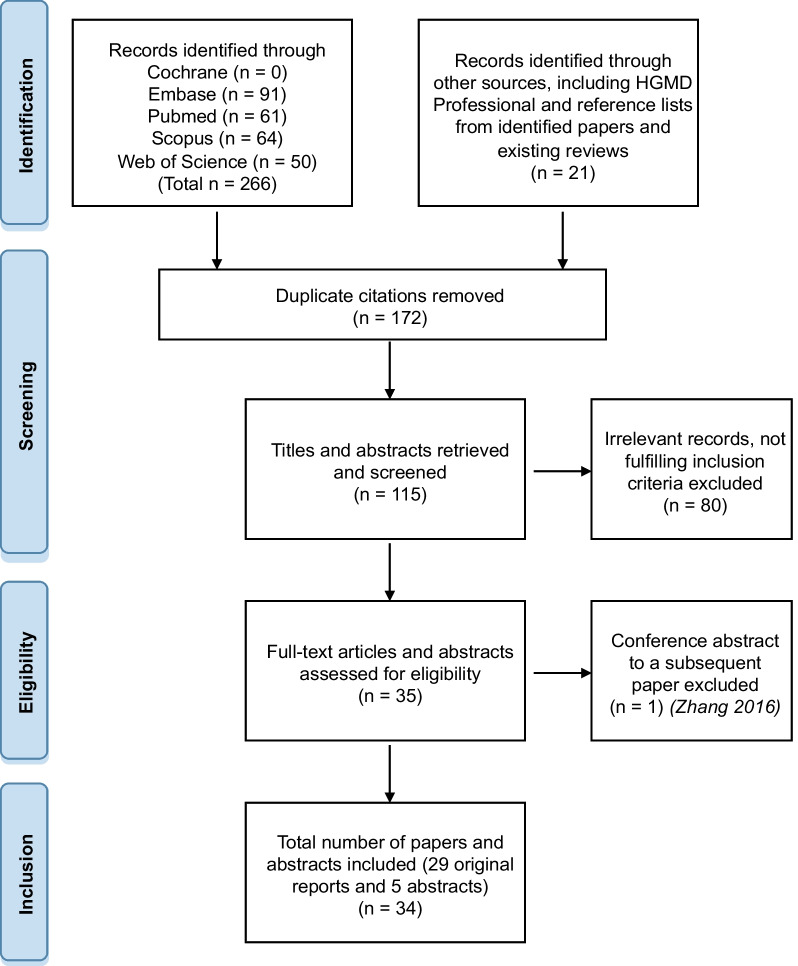
Flow diagram of inclusion of literature. A structured literature search for the part of the review concerning the spectrum of allelic variants in *SLC34A2* was performed according to preferred reporting items for systematic reviews and meta-analyses (PRISMA) 2009 guidelines [10]

The literature search yielded a total of 287 citations. Removal of duplicates, resulted in a total of 115 citations for possible inclusion. Titles and abstracts of these citations were screened by one reviewer to remove obviously irrelevant studies. One Japanese report with no abstract in English, French, or German was excluded. A conference abstract with a subsequent publication from the same authors regarding the same patients was also excluded. A total of 34 studies, including 29 original reports and five abstracts (four conference abstracts and one English abstract of a Chinese-language study), were included in the genetic part of the review (Fig. 1).

## Diagnosis, clinical characteristics and treatment

**Fig. 2:**
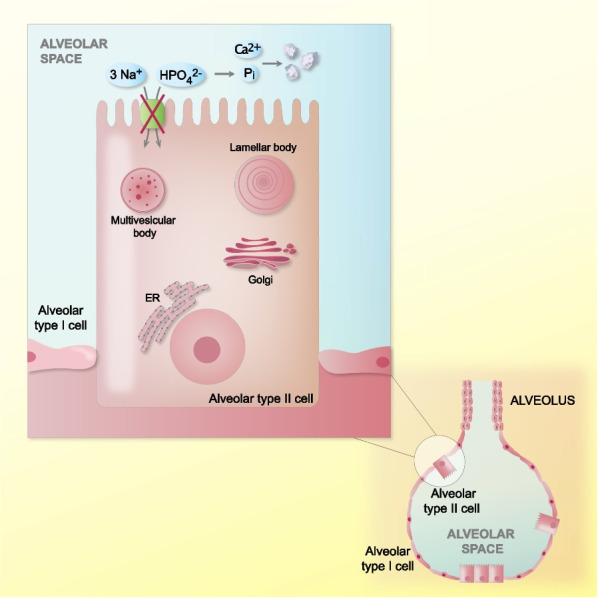
Presumed pathophysiology of PAM. Alveolar type II cell in the alveolus of the lung. Dysfunctional sodium-phosphate co-transporter (NaPi-2b) in the apical membrane leading to a decreased cell uptake of phosphate from the alveolar space and deposition of calcium-phosphate stones (microliths) due to chelation

Currently, PAM is diagnosed based on the typical radiographic appearance and detection of characteristic microliths in the bronchoalveolar lavage (BAL) fluid or a lung biopsy [1, 2, 9]. The microliths are comprised of calcareous concentric, laminated bodies typically less than 1 mm in diameter and predominantly formed of calcium and phosphorus [2]. Additional accompanying features are inflammation, fibrosis, and calcification of the lung interstitium [9, 12]. The pathophysiology of PAM is not yet fully understood. It has been proposed that the deposition of the microliths in the alveolus is caused by accumulation of phosphate from degraded surfactant phospholipids [6, 13]. Normally, phosphate will be cleared from the alveolar space by transport via NaPi-2b located in the apical membrane of the alveolar type II cell. When the transporter does not work properly, this leads to an excess of phosphate in the alveolar lumen with subsequent precipitation of extracellular calcium (Fig. 2) [5, 6, 13].

**Fig. 3:**
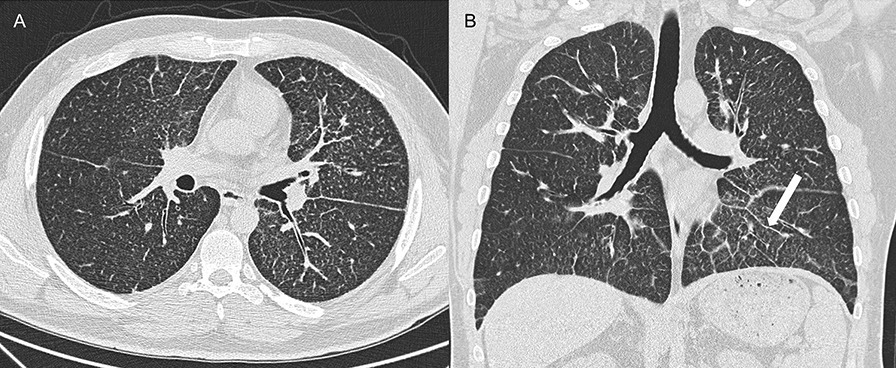
High-resolution computed tomography (HRCT) showing classical findings suggestive of pulmonary alveolar microlithiasis. **A**. Multiple microcalcifications, axial plane. **B**. Multiple microcalcifications and septal thickening (arrow), coronal plane

Although PAM is diagnosed at all ages, most patients are diagnosed between 10 and 30 years of age [9]. Many patients are diagnosed incidentally or in connection with familial investigations. Dyspnoea, dry cough, fatigue, and chest pain are frequent complains in symptomatic patients. Pneumothorax, clubbing, haemoptysis, hypoxia, and cyanosis have been reported [2, 3, 9, 14–16]. Lung function is usually normal or has a restrictive pattern [2]. PAM is generally slowly progressing, but a milder or more aggressive course might be observed [9]. The radiographic appearance is often pronounced and disproportionate to the clinical severity [15, 17]. A chest X-ray typically shows a sand-like pattern corresponding to calcifications with bilateral basal and middle zone predilection. Numerous miliary calcified nodules distributed throughout the lungs are seen on high-resolution computed tomography (HRCT) (Fig. 3) [1, 2]. The radiographic appearance is very characteristic, and in cases with typical HRCT findings, a lung biopsy is not needed to establish the diagnosis [18].

Extrapulmonary calcifications have been reported in PAM and may reflect a syndrome rather than a restricted lung disease [2, 3, 19–28]. Although the frequency of extrapulmonary manifestations is unknown, it is reasonable to hypothesize that this is not an uncommon finding as *SLC34A2* is expressed in tissues other than lung tissue [23, 29–31].

To date, no effective treatment exists except lung transplantation [9]. A few case studies report beneficial effects of the bisphosphonate, etidronate, while others report no benefit of the treatment [2, 32–35]. Use of systemic corticosteroids is generally not considered to be effective, although symptomatic improvement has been reported in a few cases [24, 36–38]. Besides, therapeutic BAL has proven ineffective, although symptomatic improvement has been described in one case [36, 39–41]. Supplemental oxygen therapy should be considered in hypoxic patients. Long-term follow-up data in PAM are sparse and the prognosis is thus unknown. However, current data indicate a poor long-term prognosis [42]. Several environmental factors such as smoking, inhalation of snuff, repetitive lung infections, and cold weather have been proposed to negatively influence course of the disease [3, 5, 43, 44].

## Etiology

### *SLC34A2*: genetic aspects

*SLC34A2* is located on the short arm of chromosome 4 (4p15.2). It contains 13 exons, of which the first seems to exist in several alternative versions, all non-coding. *SLC34A2* encodes a protein (NaPi-2b) of 690 amino acids. The gene is highly conserved in bony vertebrates and variants are therefore likely to affect the protein functionally [7, 45–47]. The expression at the protein level has mainly been investigated in animals. In addition to lung tissue, the expression at the protein level has also been found in tissues such as the small intestine, the mammary glands, the liver, the bile duct, and in the epididymis [48–52]. In addition, *SLC34A2* is expressed on the surface of different cancer types, and is a known *ROS1* (ROS proto-oncogene 1, receptor tyrosine kinase) fusion partner in non-small cell lung cancer [53–55].

### The sodium-phosphate transporter NaPi-2b

NaPi-2b (NP_006415) is a member of the transporter family SLC34, which includes the protein isoforms NaPi-2a (encoded by *SLC34A1*) and NaPi-2c (encoded by *SLC34A3*). This protein family is essential for maintaining Pi homeostasis in the human body where regulation is mediated by the intestine (NaPi-2b) and kidney (NaPi-2a, NaPi-2c) [8, 56]. NaPi-2a and NaPi-2b are both electrogenic co-transporters with a 3:1 (Na^+^: P_i_) stoichiometry, whereas NaPi-2c is electroneutral with a 2:1 (Na^+^: P_i_) stoichiometry [57]. The crystallographic structure has not been determined for any of the family members, not even the bacterial homologs. Thus, the present knowledge of structure and function is mainly based on indirect studies on wild-type and designed variants with different biophysical and biochemical methods [8]. The SLC34 group of eukaryotic transporters is presumed to have identical transmembrane (TM) topology [58–60]. The predicted topological model of the isoforms consists of 12 TM domains including two inverted repeated regions, a large extracellular loop with two N-glycosylation sites and a disulfide bridge linking the two halves of the protein, and with both C- and N-terminal regions located intracellularly. The TM domains 3–4 and 8–9 are presumed to form a substrate coordination site. Important areas for regulation and targeting are located at the C-terminal region and in the area between TM domain 10 and 11. A critical region for electrogenicity is located between TM domain 4 and 5 [8]. Recently, a three-dimensional structural model has been developed of the human NaPi-2 with the topology of the bacterial dicarboxylate co-transporter VcINDY as a template [61, 62].

### Regulation of NaPi-2b expression

NaPi-2b expression is regulated by several factors (reviewed in Hernando et al. 2018 [63]). The expression in the intestine depends on dietary Pi levels with an increased level of expression in the intestinal epithelia when the dietary levels decrease [64, 65]. Interestingly, the expression of NaPi-2b in the alveolar type II cells is seemingly not influenced by dietary intake of phosphate [48]*.* In the intestine, NaPi-2b expression is up-regulated by estrogen, vitamin D_3,_ and during metabolic acidosis, and the expression is suppressed by glucocorticoids, epidermal growth factor (EGF), and when the vitamin D receptor (VDR) is lacking [64, 66–70]. In addition, dexamethasone has been shown to down-regulate mRNA expression of NaPi-2b and decrease the uptake of phosphate in cultured alveolar type II cells from rats [71]. Contrary to this, NaPi-2b in rat lung was found not to be regulated at the mRNA level by the vitamin D analog ED-71 (1α, 25-dihydroxy-2ß-(3-hydroxypropoxy) vitamin D_3_) [72].

## The spectrum of allelic variants in PAM

**Fig. 4:**
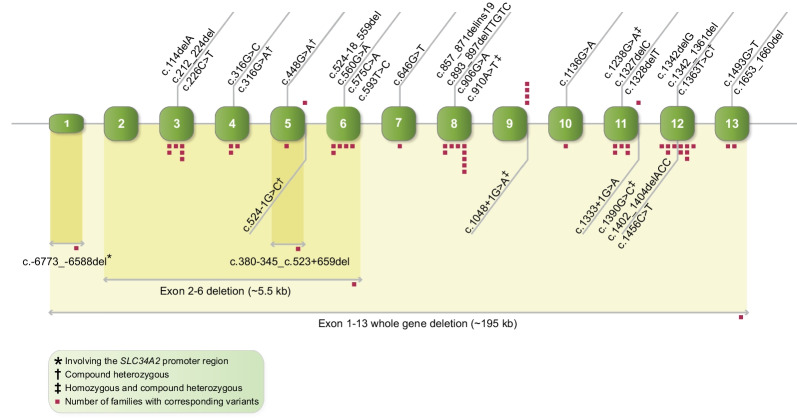
Allelic variants in *SLC34A2* in PAM patients reported in the literature [2, 3, 5, 6, 35, 43, 73–94]. Red small squares represent the number of families in which the individual variants are found. Narrow box for non-coding exon and wider box for coding exon. Exons, introns, and deletions are not drawn to scale. Variants are present in homozygous form unless otherwise stated

In 2006, variants in *SLC34A2* were initially identified as causative for PAM [5, 6]. Since then, 34 allelic variants have been documented in the literature in at least 68 patients (49 families) [2, 3, 5, 6, 35, 43, 73–94] (Fig. 4, Table 1). Only around 5% of the patients reported have been genetically investigated. However, pathogenic allelic variants in *SLC34A2* were found in more than 95% of these patients or families. In three siblings with PAM, a variant was reported in exon 2 within a sequence that, to the best of our knowledge, is not located in the coding regions of *SLC34A2* [102]. Thus, this variant is not further included in this review. Genetically unresolved cases have been reported and reports have been published on a few patients without variants in *SLC34A2* [103–105]. In one of these patients, only one pathogenic variant on a single allele was reported [105]. In addition, a cytogenetic study in a patient with myelofibrosis revealed a rearrangement of the long arms of chromosomes 4 and 5; this patient was subsequently diagnosed with PAM [106]. More efforts must be made to clarify which genetic alterations contribute to disease in these patients as the method chosen to analyse *SLC34A2* may not have been sufficient. If the genetic region sequenced is restricted only to the coding part and intron–exon boundaries, variants in introns or in the promoter region may be overlooked. In addition, the detection of larger deletions requires another analytic approach.

**Table 1:** Summary of 49 families/68 patients carrying pathogenic variants in *SLC34A2* and variant properties

Id	Allele state	Exon	Nucleotide change	Protein change	Localisation in protein^*^	Pathogenic in silico predictions^†^	Refs
1–6	Hom	1	c.-6773_-6588del	p.?	Involving promoter region and Ex. 1	NA	[5]
7	Hom	2–6	∼ 5.5 kb deletion	p.?	N-terminal to small IC-loop btw. TMDs 4–5	NA	[73]
8–9	Hom	3	c.114delA	p.Asp39IlefsTer7	N-terminal	Yes	[5]
10	Hom	3	c.212_224del	p.Asn71IlefsTer27^‡^	N-terminal	NA	[74]
11–16	Hom	3	c.226C>T	p.Gln76Ter	N-terminal	Yes	[5, 35, 75]
17–18	Hom	4	c.316G>C	p.Gly106Arg	TMD 1	Yes	[5, 76]
19	Het Het	4 11	c.316G>A c.1238G>A	p.Gly106Arg p.Trp413Ter	TMD 1 TMD 7	Yes Yes	[3]
20–21	Hom	5	c.380-345_ c.523+659del	p.?	Small EC-loop btw. TMDs 1–2 to part of TMD 3	NA	[86]
22	Het Het	5 Int. 5	c.448G>A c.524-1G>C	p.Gly150Arg p.?	TMD 2 Acceptor-splice site	Yes Yes^††^	[94]
23–24	Hom	Int. 5-Ex. 6	c.524-18_559del	p.?	TMDs 3–4	NA	[87, 92]
25	Hom	6	c.560G>A	p.Gly187Glu	TMD 4	Yes	[3]
26–28	Hom	6	c.575C>A	p.Thr192Lys	TMD 4	Yes	[43]
29–31	Hom	6	c.593T>C	p.Ile198Thr	TMD 4	Yes^§^	[77, 95]
32	Hom	7	c.646G>T	p.Gly216Ter	Small IC-loop btw. TMDs 4–5^ l^	Yes	[3]
33–34	Hom	8	c.857_871delins19	p.Ile286LysfsTer29	Large EC-loop	NA	[6]
35	Hom	8	c.893_897delTTGTC	p.Leu298GlnfsTer14	Large EC-loop	NA	[78]
36	Hom	8	c.906G>A	p.Trp302Ter	Large EC-loop	Yes	[3]
37–42	Hom	8	c.910A>T	p.Lys304Ter	Large EC-loop	Yes	[80, 83, 88]
43	Het Het	8 12	c.910A>T c.1363T>C	p.Lys304Ter p.Tyr455His	Large EC-loop Small IC-loop btw. TMDs 8–9	Yes Yes	[81]
44–47	Hom	Int. 9	c.1048+1G>A^**^	p.?	Donor-splice site	Yes^††^	[6]
48	Het Het	Int. 9 12	c.1048+1G>A c.1390G>C	p.? p.Gly464Arg	Donor-splice site TMD 9	Yes^††^ Yes	[79]
49	Hom	10	c.1136G>A	p.Cys379Tyr	TMD 6	Yes	[3]
50	Hom	11	c.1238G>A	p.Trp413Ter	TMD 7	Yes	[3]
51–52	Hom	11	c.1327delC	p.Leu443Ter	TMD 8	Yes	[3]
53–54	Hom	11	c.1328delT	p.Leu443ArgfsTer6	TMD 8	Yes	[5, 89]
55	Hom	Int. 11	c.1333+1G>A	p.?	Donor-splice site	Yes^††^	[3]
56	Hom	12	c.1342delG	p.Val448Ter	TMD 8	Yes	[5]
57–58	Hom	12	c.1342_1361del	p.Val448LeufsTer209	TMD 8	NA	[85, 91]
59–60	Hom	12	c.1390G>C	p.Gly464Arg	TMD 9	Yes	[3]
61–63	Hom	12	c.1402_1404delACC	p.Thr468del	TMD 9	Yes	[2, 3]
64	Hom	12	c.1456C>T	p.Gln486Ter	Small EC-loop btw. TMDs 9–10	Yes	[82]
65–66	Hom	13	c.1493G>T	p.Gly498Val	TMD 10	Yes	[93]
67	Hom	13	c.1653_1660del	p.Trp552AlafsTer80^‡‡^	Small EC-loop btw. TMDs 11–12	NA	[90]
68	Hom	1–13	Whole gene deletion	NA	NA	NA	[84]

*btw.* between, *Het* compound heterozygous, *EC* extracellular, *Hom* homozygous, *IC* intracellular, *Id.* patient identification, *Int.* intron, *NA* not applicable, *Ref.* reference, *TMD* transmembrane domain. ^*^A model of NaPi-2b was made by superimposing NaPi-2b on rat NaPi-2a predicted topology, modified from Forster et al. 2013 [8] and Virkki et al. 2007 [96] was used to predict the protein locations of the variants (Fig. 6), ^†^Variants were predicted to be disease causing, possibly or probably damaging or deleterious by at least one of following: Mutation Taster [97], PANTHER [98], Polyphen-2 [99], PROVEAN [100], and Human Splicing Finder [101]. ^‡^Originally reported as p.Asn71IlefsX25 [74], ^§^Prediction by PANTHER: "probably benign", ^l^Assumed critical area for electrogenicity, ^**^Originally reported as IVS8 + 1G>A [6], ^††^Prediction by Human Splicing Finder: "alteration of the WT acceptor/donor site, most probably affecting splicing". ^‡‡^Originally reported as p.Trp552AlafsTer109 [90]. *SLC34A2* DNA ref sequence: Ensembl Transcript ID ENST00000382051.8 (GRCh38.p13 assembly)

**Fig. 5:**
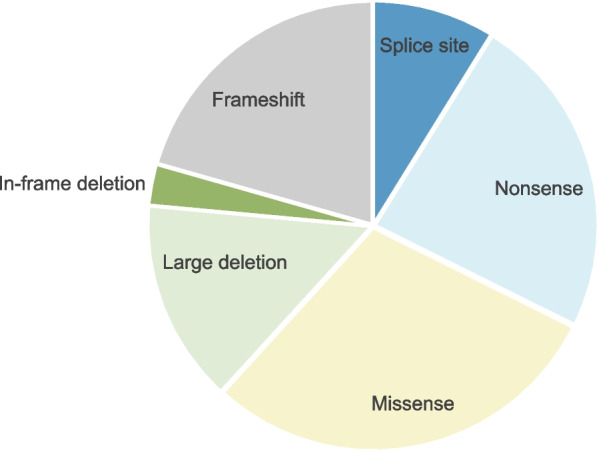
Types of allelic variants reported in PAM. Splice site 9% (3 variants), nonsense 24% (8 variants), missense 29% (10 variants), large deletion 15% (5 variants), in-frame deletion 3% (1 variant), and frameshift 21% (7 variants)

**Fig. 6:**
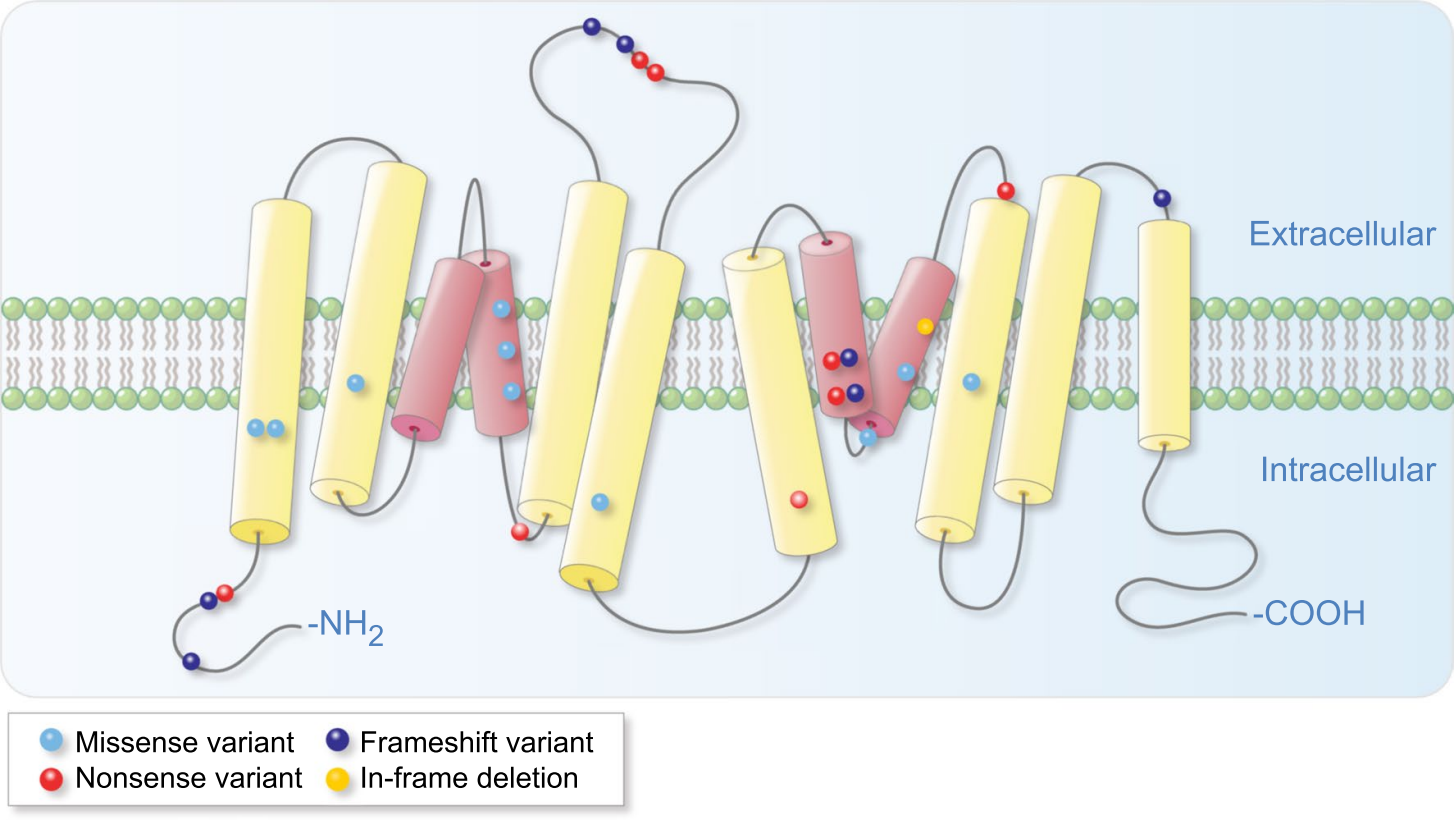
Allelic variants in *SLC34A2* in the literature marked on a model of NaPi-2b. Splice site variants and larger deletions (c.-6773_-6588del, the 5.5 kb deletion involving exons 2–6, c.380-345_ c.523+659del, c.524-18_559del, and the whole gene deletion) are not included in the figure. All variants are shown in the figure as dots. Light blue: missense variant, red: nonsense variant, dark blue: frameshift variant, yellow: in-frame deletion. The transmembrane (TM) domains with red color (TM domains 3–4 and 8–9) form the substrate coordination site. Areas for electrogenicity, regulation and targeting are found in the area between TM domains 4–5, 10–11, and at the C-terminal region [8, 107]. The model is made by superimposing human NaPi-2b on rat NaPi-2a predicted topology and is modified from Forster et al. 2013 [8] and Virkki et al. 2007 [96]. The protein sequences used for alignment in Clustal Omega version 1.2.4 [108]: Ensembl Transcript ID ENST00000382051.8 (Human (GRCh38.p13) assembly) and Ensembl Transcript ID ENSRNOT00000033749.6 (Rat (Rnor_6.0) assembly)

We performed an evaluation of the allelic variants previously reported in several standard computational prediction tools [97–101]. All analyzable variants were predicted to be deleterious by at least one prediction tool, which further supports the pathogenicity of the variants (Table 1). Five larger deletions are reported including whole gene deletion, a deletion spanning exon 2–6, a deletion including exon 5, a deletion spanning the last part of intron 5 and the first third of exon 6, and a 186-nucleotide deletion involving the promoter and exon 1. In addition, three splice site variants have been found in intron 5, 9 and in intron 11, respectively. Splice site variants and larger deletions most likely lead to loss of function or truncation of the protein with a decreased protein activity. Most variants (15/34) are either nonsense or frameshifts (Fig. 5), resulting in premature termination of the protein or probable decay of the mRNA, subsequently without any protein formation. The variants are distributed throughout the entire gene. Half of the missense variants and an in-frame deletion are located in presumably functionally important areas of the protein, likely leading to protein damage (Fig. 6).

Exon 12 is most frequently involved, and almost one-third (10/34) of the allelic variants are found in the same genomic area within 129 nucleotides in exons 11–12 and intron 11. Two variants (c.1402_1404delACC and c.1390G>C) are located in or nearby four 3-nucleotide (ACC) tandem repeats, which may predispose replication errors. Furthermore, two allelic variants (c.316G>C and c.316G>A) result in the same change at the protein level (p.Gly106Arg), and two variants (c.1327delC and c.1328delT) affect another amino acid (Leu443). The amino acid positions 106 and 443 may therefore represent other hot spots for pathogenic variants in NaPi-2b.

In almost all patients, the identified variants were in the homozygous state. Only four cases are described with variants in the compound heterozygous state (combining c.316G>A and c.1238G>A, c.448G>A and c.524-1G>C, c.910A>T and c.1363T>C, and c.1048 + 1G>A and c.1390G>C) [3, 79, 81, 94]. Strikingly, these combinations of variants consist of a missense variant with either nonsense or a splice site variant on the other allele. Some of these variants have previously been described in a homozygous state in several patients [3, 6, 80, 83, 109]. A patient was reported with only one pathogenic allelic variant [105], which alone is unlikely to explain the genetic cause of the disease since PAM is considered to follow a recessive inheritance pattern and disease has never been reported in carriers.

So far, the majority (22/34) of the allelic variants have only been reported in a single patient or in one family. Four variants (c.226C>T, c.910A>T, c.1048 + 1G>A, and c.1402_1404delACC) have been described in three to five unrelated patients/families (Fig. 4, Table 2). So far, c.226C>T is only found in patients of Middle Eastern origin, c.910A>T in Chinese patients, c.1048 + 1G>A in Japanese patients, and c.1402_1404delACC in patients of European origin.

**Table 2:** Demographics, symptoms, and smoking status in patients with *SLC34A2* variants

*SLC34A2* variant	Patients	Sex, Age (yrs.) in report^*^/at diagnosis	Origin	Cons	Symptoms (Age at debut of symptoms)	Smoking	Other	Refs
c.-6773_-6588del^†^	1 (uncle) 2 (sib 1/2) 3 (sib 2/2) 4 (sib 1/3) 5 (sib 2/3) 6 (sib 3/3)	M, 34/25 M, 17/7 M, 13/3 M, 17/11 M, 15/9 M, 11/5	TUR	Yes	Gradual decrease in exercise tolerance and dyspnoea (25) Yes, not specified (7) Yes, not specified (3) No No No	Yes Yes No No No No		[5, 110]
5.5 kb deletion^†^	7	F, 56/56	JAP	Yes	Progressive dyspnoea (−)	–	PH	[73]
c.114delA^†^	8	F, 20/9	TUR	–	Growth retardation as child. No clinical findings at age 20 (9)	No		[5, 32]
	9	F, 38/–	TUR	–	No	No		[5]
c.212_224del^†^	10	F, 58/58	ITA	Yes	No	No	Sister with PAM	[74]
c.226C>T^†^	11	F, 35/–	TUR	–	Yes, not specified (29)	No		[5]
	12 (twin 1) 13 (twin 2)	F, 5/5 F, 5/5	CAN	Yes	No No	– –	Middle-Eastern desc	[75]
	14 (sib 1) 15 (sib 2) 16 (sib 3)	M, 11/11 F, 4/4 F, 4/4	TUR	– – –	No – –	– – –		[35]
c.316G>C^†^	17	F, 27/3.5	TUR	No	Fatigue, cough, and exertional dyspnoea as child (~ 2.5). Normal physical findings and exercise capacity at age 27	No		[5, 32, 111]
	18	F, 43/43	TUR	–	Exertional dyspnoea, cough (−)	–	Suspicious familial history of PAM	[76]
c.316G>A^‡^ + c.1238G>A^‡^	19	F, 39/22	USA	Yes	Dyspnoea, chest pain (−)	No		[3]
c.380-345_c.523+659del^†^	20 (sib 1) 21 (sib 2)	F, 40/40 F, (adult)^§^/ –	CHI	Yes	Progressive dyspnoea (37) No	No –		[86]
c.448G>A + c.524-1G>C	22	M, 8/ –	CHI	No	No	–		[94]
c.524-18_559del^†^	23	M, 1/1	UGA	Yes	Progressive dyspnoea developing to severe respiratory distress and hypoxaemia (2 mos.)	–	Adopted	[87]
	24	F, 16/16	EAF	–	Recurrent dizziness, occasional cough upon physical stress and epigastric pain (−)	–		[92]
c.560G>A^†^	25	F, 9/5	SPA	Yes	No	–		[3]
c.575C>A^†^	26 (sib 1) 27 (sib 2) 28 (sib 3)	M, 53/– M, 40/– F, 49/–	CHI	Yes	No No Dyspnoea, irritable cough (−)	No No No	PH	[43]
c.593 T>C^†^	29 (sib 1) 30 (sib 2) 31 (sib 3)	M, 41/41 M, 23/23 M, 23/23	LIB	–	No No No	No – –		[77, 95]
c.646G>T^†^	32	F, –/66	ITA	–	Dyspnoea, cough, asthenia (−)	No	PH, PAM in relatives	[3]
c.857_871delins19^†^	33	F, –	JAP	Yes^l^	–	–	PAM in family members	[6]
	34	F, 43/10	JAP	–	Dyspnoea, anorexia (−)	–	Deceased	[6, 112]
c.893_897delTTGTC^†^	35	M, 38	ISR	–	Exertional dyspnoea, cough, haemoptysis (35)	Yes		[78]
c.906G>A^†^	36	F, 40/34	FRA	No	Dyspnoea (−)	No	Moroccan desc., siblings with PAM	[3]
c.910A>T^†^	37 (sib 1) 38 (sib 2)	–, – –, –	CHI	–	– –	– –		[83]
	39	M, > 55/25	CHI	Yes	Exertional dyspnoea, cough (33)	No	Sisters with PAM	[80]
	40	F, 42/20	CHI	No	Exertional dyspnoea (in her 40s)	No		[80]
	41 (sib 1) 42 (sib 2)	F, 52/– F, 39/–	CHI	Yes	Both sisters had recurrent cough, progressive dyspnoea (−)	No No		[88]
c.910A>T^‡^ + c.1363T>C^‡^	43	M, 43/43	CHI	No	Dyspnoea, chest tightness (42)	Yes	PH	[81]
c.1048+1G>A^†^	44 (sib 1) 45 (sib 2)	F, – F, – (adult)	JAP	Yes	– –	– –	Deceased	[6, 113]
	46	M, -	JAP	Yes	–	–	PAM in family members	[6]
	47	F, –	JAP	–	–	–	Deceased, PAM in family members	[6]
c.1048+1G>A^‡^ + c.1390G>C^‡^	48	F, 28/27	JAP	No	No	–		[79]
c.1136G>A^†^	49	M, 54/46	ITA	No	No	No		[3]
c.1238G>A^†^	50	F, 37/–	USA	Yes	Dyspnoea, cough (−)	–		[3]
c.1327delC^†^	51 (sib 1) 52 (sib 2)	F, 47/20 F, 52/23	NOR	No	Dyspnoea, chest pain, asthenia (−) Dyspnoea, cough, chest pain, asthenia (−)	ES ES	PH, deceased	[3]
c.1328delT^†^	53	M, 24/–	TUR	–	Yes, not specified (21)	No	Deceased	[5]
	54	F, 27/27	MOR	Yes	Exertional dyspnoea, cough (22)	–		[89]
c.1333+1G>A^†^	55	M, 58/19	USA	–	Dyspnoea, cough (−)	ES	PH	[3]
c.1342delG^†^	56	M, 39/–	TUR	–	Yes, not specified (26)	No		[5]
c.1342_1361del^†^	57^**^	–, –	–	–	–	–		[85]
	58	M, 9/1	MOR	–	Acute respiratory episodes, decreased chest expansion, exercise-induced dyspnoea, chest pain (4 mos.)	–	Adopted	[91]
c.1390G>C^†^	59 (sib 1) 60 (sib 2)	F, 14/5 M, 9/9 mos	SPA	No	Both siblings had pneumonias and broncho-obstructive crises until age 4 yrs., asymptomatic hereafter	– –		[3]
c.1402_1404delACC^†^	61	M, 32/16	DEN	–	Dyspnoea, cough, chest pain, asthenia (−)	Yes		[2, 3]
	62	M, 62/50	DEN	No	Dyspnoea, asthenia (−)	Yes		[2, 3]
	63	F, 69/51	USA	–	Dyspnoea, cough, asthenia (−)	ES	PH, Italian desc	[3]
c.1456C>T^†^	64	F, 12/12	TUR	Yes	No	–	Sister with PAM	[82]
c.1493G>T	65 (sib 1) 66 (sib 2)	M, 23/23 F, 18/18	BAR	No	Fever and productive cough of 2-day duration (23) No	No –		[93]
c.1653_1660del	67	F, 45/45	UK	Yes	Dry cough (−)	–		[90]
Whole gene deletion^†^	68	F, 20	MOR	Yes	Exertional dyspnoea (−)	No		[84]

*CAN* Canada, *CHI* China, *Cons.* Consanguinity, *DEN* Denmark, *UK* United Kingdom, *desc.* descent, *ES* Ex-smoker, *FRA* France, *ISR* Israel, *ITA* Italy, *JAP* Japan, *LIB* Libya, *MOR* Morocco, *NOR* Norway, *PH* Pulmonary hypertension, *TUR* Turkey, *sib* sibling, *SPA* Spain, *U* Uncertain, *UGA* Uganda, *EAF* East Africa, *BAR* Bahrain,—Not stated or not relevant. ^*^Age in the latest report if patient is reported in more than one paper. ^†^Homozygous. ^‡^Heterozygous. ^§^Assumed as being adult as her sister was 40 yrs old. ^l^Huqun et al. assumed the presence of consanguinity. ^**^Number of patients in the original report is not stated. *SLC34A2* DNA reference sequence: Ensembl Transcript ID ENST00000382051.8 (GRCh38.p13 assembly)

### Demographics and clinical data in patients with *SLC34A2* variants

**Fig. 7:**
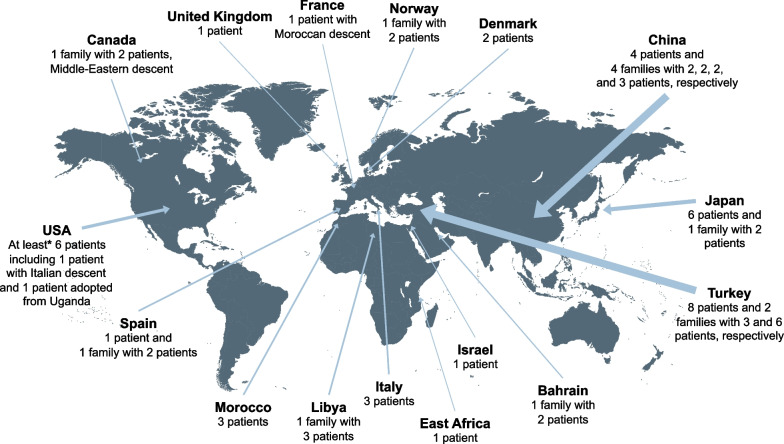
Documented PAM cases with known *SLC34A2* variants [2, 3, 5, 6, 43, 73, 74, 77–82, 84–86, 88–94]. *In one American report, no information was available regarding country of origin and number of patients [85]. The thickness of the arrows is proportional with the number of patients

Generally, patients reported with *SLC34A2* variants present with typical features of PAM, including e.g., variability in age, symptoms, and clinical findings, although detailed clinical data are missing in many reports. Table 2 summarizes patient demographics, symptoms, and smoking status. Patients reported with variants come from countries all over the world, and most were adults (Fig. 7). The age span was 9 months to 69 years with a slight female predominance. The presence of variants was a consequence of consanguineous marriages in 63% of the families. This may be an underestimation as there was no information on consanguinity in approximately 40% of the families.

Twenty-two patients were children at time of diagnosis, and they were most often diagnosed in a familial setting or incidentally, and the diagnosis was almost exclusively based on BAL or biopsy. Approximately half of the children (9/19) were asymptomatic with normal lung function (60% (9/15)). Radiological abnormalities were reported in all children, but only around half of the reports described calcifications. Among the reports including information on age, 68% (26/38) of the adults were symptomatic. Almost 90% (22/25) of the adults had abnormal lung function. All reported radiological findings were typical for PAM and in around 20% (7/32), the diagnosis was based on radiographic findings only.

## Genotype–phenotype correlation

It remains to be explored whether there is a genotype–phenotype correlation in PAM. Functional studies exploring the effect of human *SLC34A2* variants are sparse, and there is no standardized criterion for clinical classification. In our recent report including 14 PAM patients, an association between disease and variant severity was found. Although an association was found, we highlight the challenge of proper classification of disease and variants and the need for confirmation in a larger number of patients [3]. Only a few case reports have been published describing patients and families with recurrent variants (Table 2). Generally, it is difficult to compare the clinical data in the case reports as the descriptions are not standardized. Furthermore, the age of the patients varied considerably and asymptomatic children and young adults may develop symptoms later in life, which complicates the phenotype evaluation in these patients. Even though data are scarce, smoking might be associated with more severe disease [3, 5, 78, 81].

## Functional studies of *SLC34A2* variants

Two *SLC34A2* variants identified in six Japanese patients (c.1048 + 1G>A (p.?) and c.857_871delins19 (p.Ile286LysfsTer29) were found to impair phosphate transport in the presence of sodium when expressed in *Xenopus laevis* oocytes [6]. Recently, our group investigated four *SLC34A2* variants previously reported in PAM patients (c.910A>T (p.Lys304Ter), c.1328delT (p.Leu443ArgfsTer6), c.1402_1404delACC (p.Thr468del), and c.1456C>T (p.Gln486Ter)). NaPi-2b mutant constructs were expressed in *Xenopus laevis* oocytes, and transport function was investigated with a ^32^Pi uptake assay. All mutants were found non-functional [114]. Interestingly, two previous studies of the rat and the human NaPi-2a expressed in *Xenopus laevis* oocytes included mutants at the same amino acid positions as c.1390G>C (p.Gly464Arg) and c.1402_1404delACC (p.Thr468del), which was later described in PAM patients [62, 115]. Amino acid substitutions with cysteine, using the substituted cysteine accessibility method (SCAM) [115] or alanine substitution [62] revealed non-functional mutants, except when the threonine corresponding to Thr467 in human NaPi-2b was substituted with cysteine. In addition, the variant c.575C>A (p.Thr192Lys) found in a Chinese family was investigated in human alveolar epithelial cells (A549 cells) and revealed signs of reduced phosphate transport function compared to normal controls [116]. Generally, data from these reports support the underlying dysfunction of NaPi-2b in PAM.

## Animal models in PAM

Several conditional *Slc34a2* knock-out (KO) models have been developed and have provided important knowledge of possible compensatory mechanisms of lost active Na^+^-dependent phosphate transport [51, 52, 117, 118]. In a study with a conditional *Slc34a2* KO mouse model in the lung epithelium, a PAM phenotype with progressive radiographic lung manifestations including microlith accumulation, inflammation, and fibrosis was reported. The *Slc34a2* KO mice showed no clear compensatory up-regulation of other sodium-phosphate co-transporters. However, expression of the sodium-dependent phosphate transporter Pit-1 (*Slc20a1*) was found slightly increased in alveolar type II cells of *Slc34a2* KO mice on a low-phosphate diet. There was also evidence of microlith burden reduction in the mice during phosphate-restrictive diet. When measuring levels of calcium, phosphate, total protein, SP-D, and saturated phosphatidylcholine, which is a major component of pulmonary surfactant, all the parameters were increased in the BAL fluid of *Slc34a2* KO mice compared to normal mice. Furthermore, serum SP-D and inflammatory mediating cytokine MCP-1 (monocyte chemotactic protein 1) were higher in NaPi-2b deficient mice compared to control mice, and it increased with the progression of microlith deposition. A month after microliths from *Slc34a2* KO mice were instilled into the lungs of normal mice, the microcalcifications cleared completely, without any evidence of inflammation or fibrosis. The serum level of MCP-1 in these mice reached baseline at the end of the time-period suggesting MCP-1 as a potential biomarker of disease burden. Based on data from this study, the authors concluded that gene editing of NaPi-IIb expression in the lung may be a promising future therapeutic strategy in PAM [118].

## Current gaps in understanding of PAM

The discovery of *SLC34A2* as the causative gene in PAM has brought us a step closer to understand this heterogeneous disease, although the pathophysiology is not yet clear.

Further studies, including investigations of pathogenic variants in *SLC34A2* in cells and animal models, are needed to explore the basic mechanisms of the disease. Investigation of underlying factors, including possible compensatory mechanisms such as mediation of phosphate by other transporters in the alveolar type II cell, is necessary and possible involvement of environmental factors should be explored. Furthermore, the few patients without *SLC34A2* variants should be further evaluated to identify alternative genetic causes.

Except for lung transplantation, no cure or effective treatment is currently available in PAM. Neither variant-specific therapy involving e.g., systemically, or locally administered agents that could increase the quantity and the function of NaPi-2b in the lungs nor gene therapy, either via gene addition or clustered regularly interspaced short palindromic repeats (CRISPR)-based gene editing, has been tested in patients with PAM. In patients without advanced disease, gene therapy could possibly cure the patients as it is expected to be persistent in the whole lifespan of the recipient cells. You could speculate that patients with a high disease burden may not benefit from gene therapy to the same extent. In any case, detailed knowledge about the molecular consequences of the different variants identified in patients with PAM is required to be able to treat successfully based on these techniques.

To be able to develop a molecular classification, genetic testing should be performed in more patients, and the spectrum of variants should be evaluated for distinct function and distribution patterns. It is essential to explore and characterize variants in patients and compare these findings to careful clinical characterization of patients. A systematic detailed description of patient data in case reports is recommended and should, in addition to symptoms and clinical findings, include disease course, medical history, and presence of extrapulmonary calcifications, family history, consanguinity, smoking status and other possible triggers. It would indeed be desirable to have a validated disease severity classification, which would be helpful to assess disease burden, stratify patients, and to perform research. Clinical research would also benefit from an international PAM database including de-identified clinical, genetic, and demographic data.

## Genetic counseling

Genetic counseling of patients with PAM is recommended. This will provide useful information for patients and their families including the possibility of genetic testing of other family members and if relevant, the possibility of prenatal/preimplantation genetic diagnostics. In extended consanguineous families with a genetically proven case of PAM, other related couples and family members could benefit from genetic counseling. Although no cure or effective treatment is currently available except for lung transplantation, diagnosis in childhood or adolescence permits early family education and genetic counseling. In addition, it will be possible to initiate more intensive supportive care earlier, including e.g., pneumococcal and influenza vaccinations, and to plan for future transplantation.

## Conclusions

PAM is a rare genetic lung disease with a varying clinical course. The genetics of PAM, including the presence of a possible genotype–phenotype association, remains to be explored. Variants in *SLC34A2* are found in almost all patients undergoing genetic evaluation. So far, 34 allelic variants are reported in at least 68 patients, with most variants described in only a single patient. The occurrence of consanguinity is significant. We recommend a thorough systematic clinical description together with a genetic investigation in all new cases. A clinical grade system would be useful, and clinical studies and functional and experimental studies of the variants are needed to explore future treatment strategies. Finally, since the proportion of patients with *SLC34A2* variants seems to be very high, the genetic characterization may in some cases be the preferred diagnostic tool to invasive investigations, especially in the diagnostics of children.

## Data Availability

Data sharing is not applicable to this article as no datasets were generated or analyzed for the writing of this review.

## Abbreviations

PAM: Pulmonary alveolar microlithiasis
NaPi-2b: The sodium-dependent phosphate transport protein 2b
Pi: Inorganic phosphate
BAL: Bronchoalveolar lavage
CRISPR: Clustered regularly interspaced short palindromic repeats
HRCT: High-resolution computed tomography
SP: Surfactant Protein
ROS1: ROS proto-oncogene 1; receptor tyrosine kinase
NaPi-2a: The sodium-dependent phosphate transport protein 2a
NaPi-2c: The sodium-dependent phosphate transport protein 2c
TM: Transmembrane
EGF: Epidermal growth factor
VDR: Vitamin D receptor
ED-71: 1α, 25-Dihydroxy-2ß-(3-hydroxypropoxy) vitamin D_3_
A549 cells: Human alveolar epithelial cells
SCAM: The substituted cysteine accessibility method
KO: Knock-out
*Slc20a1*: The sodium-dependent phosphate transporter Pit-1
MCP-1: Monocyte chemotactic protein 1
